# Needs of older adults in Kazakhstan: analysis and psychometric properties of the localized version of the EASYCare standard 2010 instrument

**DOI:** 10.3389/fpubh.2025.1487827

**Published:** 2025-02-05

**Authors:** Kerbez Kimatova, Lyudmila Yermukhanova, Dorota Talarska, Marzena Dworacka, Gulnar Sultanova, Gulzat Sarsenbayeva, Yerlan Bazargaliyev, Perizat Aitmaganbet, Aleksandra Suwalska, Katarzyna Wieczorowska-Tobis, Ian Philp, Slawomir Tobis

**Affiliations:** ^1^Department of Public Health and Healthcare, Marat Ospanov West Kazakhstan Medical University, Aktobe, Kazakhstan; ^2^Department of Preventive Medicine, Poznan University of Medical Sciences, Poznan, Poland; ^3^Department of Pharmacology, Poznan University of Medical Sciences, Poznan, Poland; ^4^Marat Ospanov West Kazakhstan Medical University, Aktobe, Kazakhstan; ^5^Social health insurance and public health department, JSC South Kazakhstan Medical Academy, Shymkent, Kazakhstan; ^6^Department of Internal Diseases №1, Marat Ospanov West Kazakhstan Medical University, Aktobe, Kazakhstan; ^7^Department of Mental Health, Chair of Psychiatry, Poznan University of Medical Sciences, Poznan, Poland; ^8^Geriatrics Unit, Chair of Palliative Medicine, Poznan University of Medical Sciences, Poznan, Poland; ^9^Department of Human Nutrition and Dietetics, Poznan University of Life Sciences, Poznan, Poland; ^10^Centre for Health Services Studies, University of Kent, Canterbury, Kent, United Kingdom; ^11^Department of Occupational Therapy, Poznan University of Medical Sciences, Poznan, Poland

**Keywords:** older adults, needs assessment, sustainable eldercare, tool validation, functional independence, EASYCare Standard 2010

## Abstract

**Background:**

Studies about the needs of older individuals in Central Asia are very sparse. Thus, this study aimed to evaluate the needs of older adults in Kazakhstan with the EASYCare Standard 2010 (EC) questionnaire.

**Methods:**

The study involved 524 participants aged 65 and older from various regions in Kazakhstan. Data were collected by trained research staff, and the participants’ needs were examined using median split with the three summarizing indexes of the EC system *(Independence score, Risk of breakdown in care,* and *Risk of falls).*

**Results:**

Subjects with primary education had approximately double odds of scoring above the median compared to those with higher education in *Independence score* (*p* < 0.01) and *Risk of breakdown in care* (*p* < 0.01). Individuals with primary education also had 60% higher odds of scoring above the *Risk of falls* scale threshold, indicating a risk in this category (*p* < 0.05). For the *Risk of falls* scores, financial situation was also significant; individuals having not enough to make ends meet had 75% higher odds than the remaining ones (*p* < 0.01).

**Conclusion:**

Our analysis highlights the importance of tailored interventions to address the unmet needs of the Kazakh population, particularly among those with lower education and those with financial concerns. The study also underscores the need for sustainable, comprehensive eldercare policies in Kazakhstan that account for the growing older population.

## 1 Introduction

The proportion of individuals aged 60 and over is increasing rapidly worldwide. A practical and adequate response to this demographic shift requires an accurate, personalized assessment of older individuals’ needs, which might help prevent the deterioration of their independence (1). All this has highlighted the importance of a multidisciplinary approach and also led to the development of instruments for a combined health and social needs assessment (2). One such tool is the EASYCare Standard 2010 (EC) system. Over the past two decades, it has been made available in languages from all WHO regions (3) and used to evaluate and identify the unmet needs of older individuals (4–6).

The EC system acts as a comprehensive instrument for older adults, addressing specific concerns and priorities related to their needs, health, and overall comfort (7, 8). This tool offers a straightforward and practical approach to assessing various aspects such as activities of daily living (ADL), instrumental activities of daily living (IADL), mental health, social interactions, and well-being.

Based on our literature review, research in Central Asia has been sparse to date, particularly in regard to the needs of older individuals. For example, a study performed on a representative sample of older individuals indicated that a strong family relationship and adherence to a traditional lifestyle were still preserved in Uzbekistan (9). In general, a strong cultural emphasis on respecting and caring for the older members of families was widely present. Older family members were typically held in high regard and treated by younger relatives with deference. This respect for older people is a traditional value that remains significant in Uzbek society, where their well-being and comfort are, as before, often prioritized within familial relationships. This cultural value is also upheld in Kazakhstani society.

Similarly to Uzbekistan, the population of Kazakhstan is still relatively young. In 2022, 8.0% of the population was aged 65 years and older, an increase from 6.7% in 2012. However, a parallel increase in the youngest age group (0–14 years of age) was also observed (25.4% in 2012 compared to 29.7% in 2022) (10). Still, according to current United Nations projections, by 2060, the population of older individuals in Kazakhstan is expected to more than double that of children under five and approach 30% of the working-age population (11). The demographic situation in Kazakhstan differs thus from those countries where the needs of older individuals are well characterized. Hence, the need patterns of older Kazakh citizens, shaped by the presence of relatively strong younger generations and culturally anchored care habits (12) may deviate from those characteristic of the Western world.

Available studies on aging in Kazakhstan focus on demographic trends (13) and quality of life (14). For the wider Central Asian region, two further publications discuss the welfare (15) and challenges faced by older adults (16). Data regarding the needs of the older population in Kazakhstan are available only partially and limited to certain aspects of caregiving and palliative care as well as its socio-economic needs (12, 17–19). Consequently, further studies are necessary to gain a comprehensive understanding of contemporary circumstances and requirements and identify the areas where more support is needed. We thus employed a multidimensional approach using the EC tool in the analysis of the needs of older people, which constitute an important factor in premises for the planning of sustainable eldercare for the future of Kazakhstani society. Beyond the methodological details, we emphasize the clinical and systemic implications of our study.

## 2 Materials and methods

This study was approved by the West Kazakhstan Marat Ospanov Medical University’s bioethical committee, Aktobe, Kazakhstan (October 14, 2020; № 8) and was funded by the Science Committee of the Ministry of Education and Science of the Republic of Kazakhstan (AP09562783).

Data were collected by trained research staff (a total of 5 people who also served as support for clarification purposes when needed) during the COVID-19 pandemic in 2020 and 2021. The collection was performed with the help of general practitioners, social workers, and nurses, whose role was limited to the recruitment of older adults from the lists of patients at outpatient clinics (convenience sample). Only individuals with full verbal contact and no cognitive disorders were invited. Twenty-one people declined to participate, citing lack of time or fear of COVID-19 infection. No financial incentive was offered to recruited subjects.

**Figure 1:**
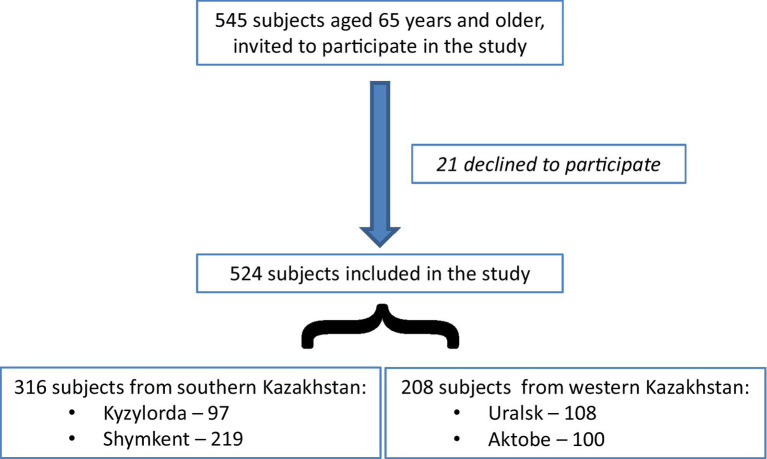
The flow chart of the study.

After participants consented by phone to participate, meetings were organized either at their homes or at outpatient clinics as convenient. Participants were informed about the study details and purpose, and written consents were obtained. The total number of participants was 524, with 97 from Kyzylorda, 219 from Shymkent (southern Kazakhstan), 108 from Uralsk (western Kazakhstan), and 100 from Aktobe (Figure 1). Data about their needs were collected using the EASYCare Standard 2010 system questionnaire (ECQ). The ECQ consists of 7 domains (49 questions) that assess the need for physical, mental, and social assistance: (1) seeing, hearing, and communicating; (2) looking after yourself; (3) getting around; (4) safety; (5) accommodation and finances; (6) staying healthy; (7) mental health and well-being.

Based on the results of the questionnaire, three summarizing indexes are calculated for each participant (20, 21):

- *Independence score* – describes a functional dependency of an individual with a score ranging between 0 and 100 points, where a lower score indicates less dependency,
- *Risk of breakdown in care* – reflects the threat of being hospitalized, with a score ranging from 0 to 12 points; a higher score indicates an increased risk of the necessity of hospitalization,
- *Risk of falls* – with a score range from 0 to 8, 3 or more points indicate an increased danger of falling.

The details on calculating the indexes have already been published (6). As there existed no Kazakh version of the ECQ, a translation from English was performed. Thereafter, the psychometric properties of the Kazakh version were evaluated in the first 100 individuals of the study. Their needs were assessed using the translated ECQ twice, 10–14 days apart. Additionally, their functional capacity in basic and instrumental activities of daily living was analyzed using gold-standard instruments: the independence in basic activities of daily living (ADL) was assessed using the Barthel Index (22) and instrumental activities of daily living (IADL) – with the Lawton Scale (23). The Barthel Index evaluates activities such as feeding, bathing, grooming, dressing, and bowel control – 10 activities in total, with final scores ranging from 0 to 100, where a lower score means greater dependency (24). The Lawton Scale assesses such activities as using the telephone, doing laundry and dressing, shopping and running errands, transportation, meal preparation, medication management, housekeeping activities, and managing finances. IADL scores range between 0 and 8 points, where lower scores indicate greater dependency.

### 2.1 Statistical analysis

STATISTICA 13.2 software (TIBCO Software, Poland) was used to perform the statistical analysis. Normality in the data distribution was examined using the Shapiro–Wilk test. Descriptive results are presented as means and standard deviations (SD), and due to the lack of normality for some data, also as medians and ranges. Participants were compared with the χ^2^ test in two age groups (65–74 and 75+) by describing socio-demographic characteristics. Since we only had eight subjects aged 85 years or older, this analysis practically reflects decade cohorts. The χ^2^ test is used to compare categorical variables.

Once calculated, the three summarizing indexes of the EC questionnaire were also analyzed with the χ^2^ test. A multiple regression model (logistic regression) was used to assess simultaneous interdependence between many variables, specifying the odds ratio and the confidence interval with a confidence limit of 95%. To divide participants according to the score in the individual indexes, a median split (splitting a continuous variable into high and low values) was used (25). This analysis was performed by comparing the subjects with the *Independence score* and the score of the *Risk of breakdown in care* results above the median to those at or below the median, and for the score of *Risk of falls –* those with increased risk to those without. All studied variables were included in multiple linear regression analysis.

Agreement between the two assessment scores on the individual items of the ECQ was checked using weighted Cohen’s kappa statistic, which is a measure of the agreement between two ordinary scaled samples and is used to compare two measurements. Its interpretation is given by Landis and Koch (26).

Cronbach’s alpha coefficient was calculated to assess internal consistency (by comparing the amount of shared variance among the individual items of a tool to the amount of overall variance), and the test–retest results were analyzed using the Wilcoxon signed-rank test (since there were two matched samples). Content validity was checked against reference instruments (ADL and IADL) with the Spearman correlation coefficient (which is a non-parametric measure of correlation between the rankings of two variables). For the interpretation of Cronbach’s alpha results, the George and Mallery rating was used (27). A *p*-value of <0.05 was considered statistically significant.

## 3 Results

### 3.1 Needs assessment

The characteristics of the study sample are presented in Table 1. It consisted of 524 individuals with a mean age of 70.3 ± 5.3 years (median 69, range 65–87), including 96 subjects aged 75 years and over (18.3%). Within the entire group, 239 were males (45.6%). Notably, almost half of the studied subjects were living with extended family (42.8%), and less than one out of four (22.7%) were living alone. Females were more frequently single (*p* < 0.001) and were more likely to live alone (*p* < 0.01) compared to males.

**Table 1:** Characteristics of studied subjects including age and gender (statistical analysis comparing younger and older subjects).

Studied parameter		Total (*n* = 524)	65–74 (*n* = 428)	75+ (*n* = 96)	
Gender	Female	285 (54.4%)	219 (51.2%)	66 (68.75%)	*p* < 0.005
	Male	239 (45.6%)	209 (48.8%)	30 (31.25%)	
Residence area	Rural	24 (4.6%)	14 (3.3%)	10 (10.4%)	*p* < 0.01
	Urban	500 (95.4%)	414 (96.7%)	86 (89.6%)	
Marital status	Single	91 (17.4%)	59 (13.8%)	32 (33.3%)	*p* < 0.0001
	Married	433 (82.6%)	369 (86.2%)	64 (66.7%)	
Living arrangements	Alone	119 (22.7%)	84 (19.6%)	35 (36.5%)	*p* < 0.0005
	With spouse	181 (34.5%)	162 (37.8%)	19 (19.8%)	
	With extended family	224 (42.7%)	182 (42.5%)	42 (43.7%)	
Education	Primary	166 (31.7%)	136 (31.8%)	30 (31.3%)	ns
	Secondary	204 (38.9%)	158 (36.9%)	46 (47.9%)	
	Higher education	154 (29.4%)	134 (31.3%)	20 (20.8%)	
Financial situation	Not enough to make ends meet	212 (40.5%)	172 (40.2%)	40 (41.7%)	ns
	At least enough to make ends meet	312 (59.5%)	256 (59.8%)	56 (58.4%)	
Are you a carer for someone?	Yes	171 (32.6%)	139 (32.5%)	32 (33.3%)	ns
	No	353 (67.4%)	289 (67.5%)	64 (66.7%)	
Does someone provide care for you?	Yes	190 (36.3%)	161 (37.6%)	29 (30.2%)	ns
	No	334 (63.4%)	267 (62.4%)	67 (69.8%)	

ns, not significant.

Based on the ECQ, the average number of needs reported by the participants was 12.1 ± 7.2 (median 11, maximum 36). Among the study participants, there was only one person who did not indicate any needs – a 70-year-old woman, single, living with an extended family.

**Figure 2:**
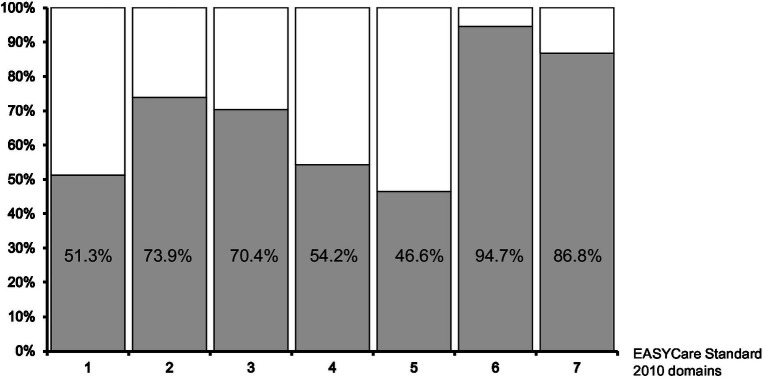
Percentages of participants with needs in the individual domains of the EASYCare 2010 questionnaire.

The majority of participants reported needs in domain 6 *(Staying healthy)* (n = 496, 94.7%, Figure 2), most frequently in response to the question “Do you take regular exercise?” – 337 individuals (64.3%) answered “No” to this question. Additionally, more women than men had concerns about their weight (156–54.7% vs. 100–41.8%; *p* < 0.01). In this domain, needs were also frequently reported for the question “Have you checked with your doctor that you are up to date with your vaccinations?” (218; 41.6%; comparably frequently in both genders; 102–35.8% vs. 116–48.5%).

In the second health-related area (domain 7, *Mental health and well-being*), 455 individuals (86.8%) reported needs. No differences in the frequency of indicated needs were found between men and women in this domain. For the majority of items in this domain, needs were reported by more than every third person: 212 subjects (40.5%) declared they were unable to pursue leisure interests, hobbies, work, and learning activities important to them, 207 participants (39.5%) complained about having trouble with sleep in the last month, 199 subjects (38.0%) reported feeling lonely, 190 (36.3%) indicated suffering from any recent loss or bereavement, 181 individuals (34.5%) had much bodily pain in the past month (114–40.0% vs. 67–28.0%; *p* < 0.01), and 179 (34.2%) reported concerns about memory loss or forgetfulness.

In domain 1 *(Seeing, hearing, and communicating)*, needs were reported by 269 subjects (51.3%); 168 individuals had vision problems (32.1%), 121 had hearing problems (23.1%), and 164 (31.3%) were unable to use the telephone independently. In domain 2 *(Looking after yourself),* needs were indicated by 387 individuals (73.9%) – almost every third person had problems with their mouth or teeth (151–28.8%), followed by housework (139–26.5%), taking their own medicine (130–24.8%), and preparing meals (117–22.3%).

In domain 3 *(Getting around)*, 369 individuals (70.4%) reported needs, including 141 (26.9%) who had issues getting to public services (66–23.2% vs. 74–31.0%; *p* < 0.05).

In domain 4 *(Safety)*, 284 subjects (54.2%) reported needs, with over a quarter indicating they lacked someone who would be able to help in case of illness or emergency (151–28.8%). In domain 5 *(Accommodation and finance)*, 244 individuals (46.6%) reported needs, with 213 (40.6%) expressing a desire for advice about financial allowances or benefits.

### 3.2 Summarizing indexes

The average *Independence score* was 10.9 ± 12.6 (median 7; range 0–75). Only 12 individuals scored above 50, which is more than half of the possible points on this scale. As many as 118 people had a score of 0.

The mean *Risk of breakdown in care* index result was 2.8 ± 2.3 (median 2, range: 0–10). On this scale, 41 individuals scored above 6, which is more than 50% of the possible points; 74 subjects had a score of 0 points.

The average *Risk of falls* index value was 1.9 ± 1.7 (median 2, range: 0–8). A score of 3 or more, indicating an increased risk of falls, was found in 179 individuals (34.2%).

A binary logistic regression analysis of determinants for the number of needs above the median for the first two summary indexes showed their relationship with education level. Participants with primary education had approximately double odds of scoring above the median compared to those with higher education in *Independence score* (*p* < 0.01) and *Risk of breakdown in care* (p < 0.01). Individuals with primary education also had 60% higher odds of scoring above the *Risk of falls* scale threshold, indicating a risk in this category (*p* < 0.05). For the *Risk of falls* scores, financial situation was also significant; individuals having not enough to make ends meet had 75% higher odds than the remaining ones (*p* < 0.01). Tables 2–4 present the multivariable analysis of determinants of needs in the studied group. Table 5 contains the results of descriptive statistics for needs in the individual ECQ domains.

**Table 2:** Multivariable analysis of determinants for *Independence score;* odds ratios (OR) and 95% confidence intervals are presented.

Analyzed parameter		Independence score	
		*n*; percentage	OR (confidence interval)
Gender	Males	129; 54.0%	OR 0.787 (0.548–1.131) ns
	Females	144; 50.5%	
Age (years)	65–74	214; 50.0%	OR 1.558 (0.964–2.518) ns
	75+	59; 61.5%	
Residence area	Rural	15; 62.5%	OR 0.814 (0.333–1.990) ns
	Urban	258; 51.6%	
Marital status	Single	96; 55.8%	OR 0.932 (0.555–1.567) ns
	Married	177; 50.3%	
Living arrangements	Alone (I)	71; 59.7%	II vs. I: OR 0.710 (0.370–1.364) ns III vs. I: OR 0.734 (0.410–1.314) ns
	With spouse (II)	89; 49.2%	
	With extended family (III)	113; 50.5%	
Education	Primary (I)	101; 60.8%	II vs. I: OR 0.731 (0.473–1.129) ns **III vs I: OR 0.494 (0.311–0.783)** ***p* < 0.005**
	Secondary (II)	107; 52.5%	
	Higher education (III)	65; 42.2%	
Financial situation	Not enough to make ends meet	118; 55.7%	OR 0.868 (0.604–1.247) ns
	At least enough to make ends meet	155; 49.7%	
Are you a carer for someone?	Yes	89; 52.1%	OR 1.089 (0.717–1.654) ns
	No	184; 52.1%	
Does a family member/friend provide care for you?	Yes	101; 53.2%	OR 0.812 (0.539–1.222) ns
	No	172; 51.5%	

ns, not significant. Statistically significant values are printed bold.

**Table 3:** Multivariable analysis of determinants for the *Risk of breakdown in care;* odds ratios (OR) and 95% confidence intervals are presented.

Analyzed parameter		Risk of breakdown in care	
		*n*; percentage	OR (confidence interval)
Gender	Males	150; 62.8%	OR 1.091 (0.749–1.590) ns
	Females	188; 66.0%	
Age (years)	65–74	270; 63.1%	OR 1.402 (0.841–2.336) ns
	75+	68; 70.8%	
Residence area	Rural	16; 66.7%	OR 0.943 (0.379–2.347) ns
	Urban	322; 64.4%	
Marital status	Single	114; 66.3%	OR 0.794 (0.462–1.362) ns
	Married	224; 63.6%	
Living arrangements	Alone (I)	83; 69.8%	II vs. I: OR 0.707 (0.357–1.400) ns III vs. I: OR 0.740 (0.401–1.365) ns
	With spouse (II)	113; 62.4%	
	With extended family (III)	142; 63.4%	
Education	Primary (I)	120; 72.3%	II vs. I: OR 0.681 (0.428–1.084) ns **III vs I: OR 0.543 (0.336–0.880)** ***p* < 0.05**
	Secondary (II)	131; 64.2%	
	Higher education (III)	87; 56.5%	
Financial situation	Not enough to make ends meet	148; 69.8%	OR 0.730 (0.498–1.068) ns
	At least enough to make ends meet	190; 60.9%	
Are you a carer for someone?	Yes	103; 60.2%	OR 1.372 (0.890–2.116) ns
	No	235; 66.6%	
Does a family member/friend provide care for you?	Yes	121; 63.7%	OR 1.172 (0.764–1.795) ns
	No	217; 65.0%	

ns, not significant. Statistically significant values are printed bold.

**Table 4:** Multivariable analysis of determinants of *Risk of falls;* odds ratios (OR) and 95% confidence intervals are presented.

Analyzed parameter		Risk of falls	
		*n*; percentage	OR (confidence interval)
Gender	Males	73; 30.5%	OR 1.293 (0.878–1.903) ns
	Females	106; 37.2%	
Age (years)	65–74	141; 32.9%	OR 1.150 (0.701–1.885) ns
	75+	38; 39.6%	
Residence area	Rural	11; 45.8%	OR 0.889 (0.368–2.148) ns
	Urban	168; 33.6%	
Marital status	Single	77; 44.8%	OR 0.654 (0.381–1.123) ns
	Married	102; 29.0%	
Living arrangements	Alone (I)	57; 47.9%	II vs. I: OR 0.933 (0.477–1.828) ns III vs. I: OR 0.655 (0.362–1.185) ns
	With spouse (II)	59; 32.6%	
	With extended family (III)	63; 28.1%	
Education	Primary (I)	71; 42.8%	**II vs I: OR 0.625 (0.395–0.989)** ***p* < 0.05** III vs. I: OR 0.769 (0.476–1.244) ns
	Secondary (II)	59; 28.9%	
	Higher education (III)	49; 31.8%	
Financial situation	Not enough to make ends meet	89; 42.0%	**OR 0.570 (0.389–0.835)** ***p* < 0.01**
	At least enough to make ends meet	90; 28.9%	
Are you a carer for someone?	Yes	62; 36.3%	OR 0.836 (0.536–1.305) ns
	No	117; 33.1%	
Does a family member/friend provide care for you?	Yes	63; 33.2%	OR 1.040 (0.671–1.612) ns
	No	116; 34.7%	

ns, not significant. Statistically significant values are printed bold.

**Table 5:** Descriptive statistics for all ECQ domains.

EASYCare domain		Maximum number of needs	Mean ± SD (median; range)
1	Seeing, hearing, and communicating	4	1.0 ± 1.3 (1.0; 0–4)
2	Looking after yourself	13	2.1 ± 2.3 (1.0; 0–12)
3	Mobility (getting around)	8	1.8 ± 1.8 (1.0; 0–8)
4	Safety	5	1.0 ± 1.3 (1.0; 0–5)
5	Accommodation and finances	3	0.7 ± 0.9 (0.0; 0–3)
6	Staying healthy (prevention)	7	2.7 ± 1.4 (3.0; 0–7)
7	Mental health and well-being	9	2.7 ± 2.1 (2.0; 0–4)

### 3.3 The validation study

The mean age of study subjects who completed the ECQ twice (n = 100) was 70.7 ± 4.6 years (median: 70, range: 65–85). Among them, 38 were males. The validation group did not differ from the general study sample in terms of gender, residence area, marital status, financial situation, living arrangements, being a carer for someone or being a care recipient; differences were present for education only (due to the fractions of primary and higher education subgroups - 14.0% vs. 31.7 and 44.0% vs. 29.4%, respectively). The Cronbach’s alpha coefficient for the whole ECQ was 0.83.

No significant differences were found in the *Independence score*, *Risk of breakdown in care*, and *Risk of falls* between the two assessments. Cohen’s kappa coefficient across all domains ranged between 0.81 and 0.95, showing almost perfect agreement between all scale domains (Table 6).

**Table 6:** Weighted Cohen’s kappa values of the validation study for two assessments in all domains of the questionnaire.

EASYCare domain		Kappa value
1	Seeing, hearing, and communicating	0.95
2	Looking after yourself	0.90
3	Mobility (getting around)	0.87
4	Safety	0.83
5	Accommodation and finances	0.95
6	Staying healthy (prevention)	0.81
7	Mental health and well-being	0.92

The mean Barthel Index of studied subjects was 94.0 ± 10.4 (median 100; range: 45–100), and the Lawton scale–7.5 ± 1.2 (median 8, range 2–8). *Independence score* and *Risk of breakdown in care* showed a good correlation with the scores of both the Barthel Index and the Lawton scale, which are gold-standard instruments for the assessment of functional independence (Table 7). For *Risk of falls,* moderate correlation was found.

**Table 7:** Correlations between the EASYCare summarizing indexes and Barthel Index and Lawton scale.

	Barthel index	Lawton scale
Independence score	*r* = −0.94, *p* < 0.0001	*r* = −0.85, *p* < 0.0001
Risk of breakdown in care	*r* = −0.64, *p* < 0.0001	*r* = −0.54, *p* < 0.0001
Risk of falls	*r* = −0.39, *p* < 0.0001	*r* = −0.38, *p* < 0.001

## 4 Discussion

We analyzed the care needs of older adults in Kazakhstan. As far as we know, this is the first study on this topic in the whole of Central Asia. All countries in this region have a similar history – they gained independence from the Soviet Union at the end of the 20^th^ century and then undertook substantial health system reforms (28). They also share a crucial role of adherence to traditional lifestyles and strong family bonds (29), and their populations are relatively young (28). Therefore, similarities in needs and ways of their satisfaction can be expected.

Due to the lack of a standardized tool in the Kazakh language, we translated the English ECQ and validated the resulting tool. We have shown that the Kazakh version of ECQ has good to excellent psychometric properties and, therefore, can be used to assess the needs of older people.

According to our analyses, participants frequently reported needs in health-related domains (6—*Staying healthy* and 7—*Mental health and well-being*). Our previous studies on the Polish population have similarly highlighted a significant number of needs in these areas (30), which was also shown by a recent study from South Korea (31). Importantly, while the needs of older people related to healthcare are universal, there are culturally specific differences in the ways of their satisfaction (due to such factors as the presence of family support, healthcare practices or social activity patterns) (32, 33). The high prevalence of needs related to lifestyle factors (like physical activity or concerns about weight) is characteristic of other regions of the world as well (34–36). It is also noteworthy that there is a substantial need for information about vaccinations and financial support. This creates a coherent picture with necessary changes in lifestyle intertwined with the need to provide crucial information.

One-third of the respondents reported experiencing pain. The prevalence of chronic pain in older people varies greatly depending on the population studied and is expressed differently in various age subgroups (37–41). Novel approaches to pain management involving the principles of person-centered care are thus welcome (42, 43).

A substantial number of our respondents indicated that they had nobody who would be able to help them in case of illness or emergency. This is remarkable given the emphasis on the traditional family model in Kazakhstan, where nearly half of the respondents lived with extended family. This finding may, however, be influenced by the timing of the study (during the COVID-19 pandemic), as one-third of the respondents also reported suffering from recent loss or bereavement.

It is imperative to note that the ECQ inherently addresses unmet needs. Therefore, it draws attention to people with vision or hearing disorders who, despite potential corrective measures, still report difficulties. Importantly, this helps identify areas where further support is necessary. Despite a high number of subjects reporting needs, similarly to what we have previously shown for the Polish population (30), the summarizing indexes were low, indicating good functional capacity among the study participants. One may thus expect that interventions tailored for this target group will be effective. In this study, lower education was the most important determinant of poorer scores across all indexes. Analogous findings were observed in another analysis of the Polish population using the same needs assessment tool (44). In this context, a recent publication on data characterizing Kazakhstan notably stresses that, in order to support health-promoting behaviors, measures are being planned to increase the health literacy of the population (45).

In a population study from Kosovo that used the ECQ, gender has been an important determinant of the summarizing indexes (46). However, it should be noted that women differed significantly from men. Many of them had no education, and this may reflect the specific characteristics of the older population in Kosovo during the study period, almost 15 years ago.

In our investigation, a worse financial situation increased the risk of falls by the participants. This is worth noting because this factor was also shown to be significant in the earlier-mentioned paper from Kosovo (46). In our study, living alone was not observed as a significant factor for increased risk of falling. Still, living alone, particularly in an urban area, is often considered an important risk factor for falls (47, 48).

Our research has some limitations. Despite the large study group, the sample was not representative of the whole Kazakh population. The vast majority of participants lived in large cities, and there are known regional disparities in access to healthcare in Kazakhstan (49). Additionally, due to historical factors, part of the population in Kazakhstan is Russian-speaking [e.g., in West Kazakhstan, about 20%, (49)]. To generalize the results to the entire population, it is necessary to conduct studies in this demographic as well. The use of convenience sampling also means that the results reflect the needs of older people in contact with medical services rather than the general population. Furthermore, since the study is cross-sectional, it is not possible to discuss causal relationships.

A strong point of our study is addressing the needs of older adults in Central Asia, a region that is still relatively young and has strong family ties but for which demographic aging projections are similar to other regions of the world. This necessitates preparing the healthcare system; yet, among the identified seven strategic priorities to improve the efficiency and effectiveness of the healthcare system in Kazakhstan (45), none are related to aging and demographic challenges.

In an effort to establish a viable approach to problems related to aging, smart homes and Internet of Things (IoT) solutions have been recently taken into consideration in Kazakhstan (50). While the use of new technologies in sustainable geriatric care has been discussed in various contexts (51–54), it is imperative to implement them in a properly prepared way. This includes studying the needs and requirements of future users and reflecting them in the design process (55–57). The EC system can be a solid base for such an approach.

Our analysis is also meant to be a voice in the discussion on addressing the Sustainable Development Goals in care for older adults, which, so far, seems to insufficiently consider the differences in needs of various age groups across contemporary societies (58). Comprehensive assessment of the needs of older people requires dedicated tools, such as the EC system, which should be available in local languages to ensure a proper understanding of the questions posed and take into account the local context. The importance of cross-cultural adaptation is stressed in various scenarios (59, 60).

## 5 Conclusion

We showed that the Kazakh version of the ECQ demonstrated good to excellent psychometric properties, indicating its reliability and validity for assessing the needs of older people in Kazakhstan. The study found a significant number of unmet needs among older adults in Kazakhstan, particularly in areas related to health, safety, and activities of daily living. Socio-economic factors, including education level and financial situation, were significant determinants of the needs and risks among older adults: those with lower education and those with poorer financial status were particularly vulnerable to higher dependency and greater health risks. The findings suggest that tailored interventions are necessary to address the specific needs of older adults in Kazakhstan, particularly for those at higher risk of unmet needs due to lower education levels, worse financial situation, or having limited access to social and health support. The study also underscores the need for sustainable, comprehensive eldercare policies in Kazakhstan that account for the growing older population.

## Data Availability

The raw data supporting the conclusions of this article will be made available by the authors, without undue reservation.
